# Threat of Dengue to Blood Safety in Dengue-Endemic Countries

**DOI:** 10.3201/eid1501.071097

**Published:** 2009-01

**Authors:** Annelies Wilder-Smith, Lin H. Chen, Eduardo Massad, Mary E. Wilson

**Affiliations:** National University Singapore, Singapore (A. Wilder-Smith); Harvard Medical School, Boston, Massachusetts, USA (L.H. Chen, M.E. Wilson); Harvard School of Public Health, Boston (M.E. Wilson); Mount Auburn Hospital, Cambridge, Massachusetts, USA (L.H. Chen, M.E. Wilson); University of São Paulo, São Paulo, Brazil (E. Massad)

**Keywords:** Dengue, blood safety, blood transfusions, non-vector transmission, nosocomial transmission, parenteral transmission, healthcare-associated dengue, perspective

## Abstract

Dengue, the most common arbovirus infection globally, is transmitted by mosquito vectors. Healthcare-related transmission, including transmission by blood products, has been documented, although the frequency of these occurrences is unknown. Dengue is endemic to Singapore, a city-state in Asia. Using mathematical modeling, we estimated the risk for dengue-infected blood transfusions in Singapore in 2005 to be 1.625–6/10,000 blood transfusions, assuming a ratio of asymptomatic to symptomatic infections of 2:1 to 10:1. However, the level of viremia required to cause clinical dengue cases is person-dependent and unknown. Further studies are needed to establish the magnitude of the threat that dengue poses to blood safety in countries where it is endemic. It will then be possible after this information is obtained to assess whether screening is feasible and to identify approaches that are most cost-effective on the basis of characteristics of local populations and seasonality of dengue.

## Abstract 2

Modeling the risk for transmission of this disease is quantified by blood transfusions.

Emerging infectious diseases pose threats to the general human population, including recipients of blood transfusions. Dengue is an expanding problem in tropical and subtropical regions and is now the most frequent arboviral disease in the world, with an estimated annual 100 million cases of dengue fever, 250,000 cases of dengue hemorrhagic fever, and 25,000 deaths per year (*1*). Dengue virus infections have been reported in >100 countries; 2.5 billion people live in areas where dengue is endemic (*1*). It is also increasingly reported to occur in international travelers (*2*). Dengue is of major international public health concern because of the expanding geographic distribution of the virus amd competent mosquito vectors, increased frequency of epidemics, cocirculation of multiple virus serotypes, and emergence of dengue hemorrhagic fever in new areas.

Dengue virus belongs to the family *Flaviviridae* and is transmitted by mosquitoes of the genus *Aedes* (*1*). Flaviviruses are small, lipid-enveloped, positive-stranded RNA viruses (*1*). Dengue infection has a viremic phase that lasts 4–8 days (*3*), and most infections remain subclinical (*4*). Viremia can precede the onset of symptoms in persons with clinical disease. Plasma viral RNA levels range from 10^5.5^ to 10^9.3^ copies/mL, and blood collected during this phase may be infective when transfused into susceptible hosts (*5*).

A literature search conducted in March 2008 on blood transfusion and dengue did not identify any published reports of blood transfusion–associated dengue in dengue-endemic countries. Healthcare-associated transmission is difficult to ascertain in dengue-endemic countries, although there is 1 report of possible transmission of dengue in a healthcare setting in Hong Kong, Special Administrative Region, People’s Republic of China, attributed to a viremic resident of Hong Kong (*6*). In contrast, healthcare-acquired dengue infections in countries in which dengue is not endemic can be determined. Several reports have described travelers who returned from dengue-endemic countries to those not endemic for dengue and transmitted dengue infection to healthcare workers by needlestick injury or mucocutaneous exposure to blood (*7*,*8*).

Healthcare-associated transmission of viruses among humans has been recently reviewed for dengue and other flaviviruses such as West Nile virus (WNV) and yellow fever virus (*9*). Routes of transmission include percutaneous, mucous membrane, bone marrow transplant, organ transplant, hemodialysis, and transfusion of blood products (*6*–*8*,*10*–*25*) (Table) However, despite good evidence for its transmission in healthcare settings, dengue is currently not considered a risk to blood safety.

**Table:** Reported healthcare-associated transmission of flaviviruses

Virus	Route of transmission	Comment	References
Dengue	Percutaneous	Several healthcare workers were infected after needlestick injuries during care of returned travelers who had diagnoses of dengue.	(*8*,*10*–*13*)
	Mucocutaneous	A healthcare worker became infected with dengue 3 virus after being splashed in the face by blood from a febrile traveler who had a diagnosis of dengue.	(*7*,*14*)
	Blood transfusion	A 17-year-old man from Hong Kong, Special Administrative Region, People’s Republic of China, donated blood in July 2002, from which erythrocytes were transfused to a 72-year-old woman, in whom febrile illness consistent with dengue fever developed 3 d later.	(*15*)
	Bone marrow transplant	A 6-year-old child from Puerto Rico became infected with dengue 4 virus from a bone marrow transplant and died.	(*16*)
	Renal transplant	Dengue hemorrhagic fever developed after a living donor renal transplant.	(*17*)
Yellow fever	Laboratory	A laboratory technician acquired yellow fever after obtaining blood and performing a blood count on a yellow fever patient; he died subsequently. Yellow fever was transmitted to at least 30 other scientists and laboratory workers after contact with mouse or monkey blood or tissues or handling infected animals.	(*18*–*20*)
West Nile	Percutaneous	Virus was transmitted to 2 microbiologists by laceration or needlestick injuries in laboratory.	(*21*)
	Transfusion	Virus was transmitted to numerous recipients of blood products.	(*22*,*23*)
	Organ transplant	Virus was transmitted to transplant recipients from kidneys, liver, and heart of an infected donor.	(*24*)
	Hemodialysis	Virus infection in a cluster of 3 hemodialysis patients suggested transmission through a common dialysis machine.	(*25*)

We draw parallels from recent experiences with West Nile fever and encephalitis in the United States. WNV first appeared in the United States in 1999 and has since spread throughout the country, resulting in thousands of cases of disease (*26*). Approximately 80% of WNV infections are asymptomatic (*27*). By 2002, 23 patients in the United States were confirmed to have acquired WNV through transfused blood and blood products (erythrocytes, platelets, and fresh-frozen plasma) (*23*). The estimated risk for virus transmission through transfusion during the 1999 WNV epidemic in New York was 1.8/10,000–2.7/10,000 donations, and ≈2.0 viremic donations/10,000 donors in the borough of Queens (*28*). Of the 2.5 million blood donations screened for WNV from June through December 2003, 0.05% were positive at the first screening and 0.02% were confirmed (*29*). In response to these findings, by 2003, essentially all blood donations in the United States were being tested for WNV. In contrast, no screening of blood products is conducted for dengue, although dengue virus is estimated to affect >100 million persons annually in tropical and subtropical regions. A recent study in Puerto Rico reported nucleic acid testing for dengue virus in the blood supply and found the viral RNA prevalence to be 7.3/10,000 U of blood donations, which approximates the prevalence of WNV in the United States during the transmission season (*30*). In addition, screening of donors in Honduras and Brazil has identified dengue virus RNA (0.37% and 0.06% of blood donations or 37/10,000 and 6/10,000 blood donations, respectively) by using a transcription-mediated amplification assay (*31*).

We postulate that dengue virus poses a greater threat worldwide to blood safety than WNV but that this hypothesis has been neglected because dengue occurs predominantly in developing countries. We used mathematical modeling to estimate the risk for dengue in Singapore. Singapore is an industrialized Asian city-state in which dengue is endemic. This city-state has the capacity to implement blood screening for dengue. The dengue seroprevalence rate in the adult population in Singapore, which has a population of ≈4 million persons, is 45% (*32*). In 2005, 14,209 cases of symptomatic dengue infections were reported in Singapore, a large proportion (≈80%) of which were in adolescents or adults (*33*). We calculated the force of infection in this population. The force of infection is defined as follows: per capita new cases in a finite period = rate of contacts by potentially infective persons × (no. viremic persons in a population in a finite period/total population in a finite period).

If one assumes that there were 11,367 reported adult cases in Singapore in 2005 (80% of 14,209) and a ratio of asymptomatic to symptomatic cases of 2:1, a total of 34,101 viremic adults in 2005 would have viremia that lasts <1 week. This finding indicates there would be 656 infective persons per week, which translates to 0.00016 viremic persons in a finite period per total population in a finite period. If one considers that there are ≈80,000 blood transfusions in Singapore per year, there would be 1,538 transfusions per week. Therefore, the force of infection for 2005 in Singapore was ≈13 infective blood donations.

However, if we assume a ratio of asymptomatic to symptomatic cases of 10:1, we then have 125,037 viremic persons, or ≈2,405 infective persons per week. This finding indicates that the number of viremic persons in the population in a finite period/total population in a finite period is 0.00060115. We would then end up with a force of infection for 2005 of 48 infective donations. Depending on the ratio of asymptomatic to symptomatic infections, there were ≈13–48 dengue infected blood donations in Singapore in 2005. With ≈80,000 blood transfusions annually in Singapore, the proportion of infected blood transfusions would be ≈1.625–6/10,000 transfusions, which is consistent with reported dengue RNA prevalence in blood donations in Puerto Rico, Brazil, and Honduras (*30*,*31*). This proportion is similar or even higher than the estimated risk for WNV transmission by transfusion during the 1999 epidemic in New York, which was reported to be 1.8/10,000–2.7/10,000 donations (*28*). A total of 1.625–6/10,000 blood transfusions would translate to 3.25–12 potentially infective blood transfusions/1 million persons in Singapore, if one assumes a ratio of asymptomatic to symptomatic case between 2:1 and 10:1. Further, a recent report provides well-documented evidence of a cluster of blood transfusion–associated dengue infections in Singapore (*34*).

Why has transfusion-associated dengue not yet been widely recognized as a problem in dengue-endemic countries? Lack of recognition is likely due to lack of awareness that dengue is transmitted not only by vectors but also by blood products. Because of the effects of infection and recurring epidemics in dengue-endemic countries, isolated cases of healthcare-acquired infections will go unnoticed. In many healthcare facilities, patients are not protected from mosquitoes, and it is therefore difficult to ascertain whether infections were related to blood transfusions or exposure to vectors. Further, risk for transmission by transfusion may depend on the level of viremia, which has been shown to correlate with severity of disease (*35*). It is likely, although not proven, that viremia is lower and shorter in duration in asymptomatic persons than in symptomatic persons. The risk for transfusion-associated dengue will vary greatly from 1 country to another, depending on the epidemiologic pattern of dengue and the immunity level in the population. In countries where dengue is mainly a childhood disease, risk for blood transfusion–transmitted dengue will be lower because of lack of overlap of infected and blood-donating populations. However, risk for nosocomial transmission from needlesticks and other blood exposures would exist in all areas with dengue.

Blood transfusion–related dengue will likely represent only a small proportion of all dengue cases in dengue-endemic countries. Screening blood for dengue antigens in dengue-endemic countries would be costly and should therefore be recommended only after careful assessment of risk for infection and cost per blood product–associated dengue infection averted. Therefore, the first step is to quantify this risk in a systematic study. Risk will vary by geographic region and season and may change over time. We suggest targeted nucleic acid amplification testing of individual donations in high-prevalence regions, a strategy that was implemented successfully for screening of WNV in the United States in 2004 (*36*); nucleic acid amplification tests of minipools of several samples of donated blood have also been proposed (*36*). A prototype nucleic acid test, which is suitable for high-throughput screening, has been developed for detection of dengue virus RNA in blood donations (*31*). The initial study should be conducted during the dengue transmission season to identify maximum incidence of viremic donations. This testing would provide a baseline estimate of risk for transmission of infective blood. If the risk is found to be substantial, healthcare providers would need to decide at what threshold screening should be instituted. Policies will also be influenced by economic resources available and healthcare priorities of a country or region.
